# Efficacy and classification of *Sesamum indicum* linn seeds with *Rosa damascena* mill oil in uncomplicated pelvic inflammatory disease using machine learning

**DOI:** 10.3389/fchem.2024.1361980

**Published:** 2024-04-02

**Authors:** Sumbul, Arshiya Sultana, Md Belal Bin Heyat, Khaleequr Rahman, Faijan Akhtar, Saba Parveen, Mercedes Briones Urbano, Vivian Lipari, Isabel De la Torre Díez, Azmat Ali Khan, Abdul Malik

**Affiliations:** ^1^ Department of Ilmul Qabalat wa Amraze Niswan, National Institute of Unani Medicine, Ministry of AYUSH, Government of India, Bengaluru, Karnataka, India; ^2^ CenBRAIN Neurotech Center of Excellence, School of Engineering, Westlake University, Hangzhou, Zhejiang, China; ^3^ Department of Ilmul Saidla, National Institute of Unani Medicine, Ministry of AYUSH, Government of India, Bengaluru, Karnataka, India; ^4^ School of Computer Science and Engineering, University of Electronic Science and Technology of China, Chengdu, Sichuan, China; ^5^ College of Electronics and Information Engineering, Shenzhen University, Shenzhen, China; ^6^ Research Group on Foods, Nutritional Biochemistry and Health, Universidad Europea Del Atlántico, Santander, Spain; ^7^ Research Group on Foods, Nutritional Biochemistry and Health, Universidade Internacional do Cuanza, Kuito, Angola; ^8^ Research Group on Foods, Nutritional Biochemistry and Health, Universidad Internacional Iberoamericana, Arecibo, PR, United States; ^9^ Department of Signal Theory and Communications and Telemedicine Engineering, University of Valladolid, Valladolid, Spain; ^10^ Pharmaceutical Biotechnology Laboratory, Department of Pharmaceutical Chemistry, College of Pharmacy, King Saud University, Riyadh, Saudi Arabia; ^11^ Department of Pharmaceutics, College of Pharmacy, King Saud University, Riyadh, Saudi Arabia

**Keywords:** unani medicine, botanical drugs, herbal intervention, AI for medicine, drug design, female disorder, reproductive-age disorder, medical intelligence

## Abstract

**Background and objectives:** As microbes are developing resistance to antibiotics, natural, botanical drugs or traditional herbal medicine are presently being studied with an eye of great curiosity and hope. Hence, complementary and alternative treatments for uncomplicated pelvic inflammatory disease (uPID) are explored for their efficacy. Therefore, this study determined the therapeutic efficacy and safety of *Sesamum indicum* Linn seeds with *Rosa damascena* Mill Oil in uPID with standard control. Additionally, we analyzed the data with machine learning.

**Materials and methods:** We included 60 participants in a double-blind, double-dummy, randomized standard-controlled study. Participants in the Sesame and Rose oil group (SR group) (*n* = 30) received 14 days course of black sesame powder (5 gm) mixed with rose oil (10 mL) per vaginum at bedtime once daily plus placebo capsules orally. The standard group (SC), received doxycycline 100 mg twice and metronidazole 400 mg thrice orally plus placebo per vaginum for the same duration. The primary outcome was a clinical cure at post-intervention for visual analogue scale (VAS) for lower abdominal pain (LAP), and McCormack pain scale (McPS) for abdominal-pelvic tenderness. The secondary outcome included white blood cells (WBC) cells in the vaginal wet mount test, safety profile, and health-related quality of life assessed by SF-12. In addition, we used AdaBoost (AB), Naïve Bayes (NB), and Decision Tree (DT) classifiers in this study to analyze the experimental data.

**Results:** The clinical cure for LAP and McPS in the SR vs SC group was 82.85% vs 81.48% and 83.85% vs 81.60% on Day 15 respectively. On Day 15, pus cells less than 10 in the SR vs SC group were 86.6% vs 76.6% respectively. No adverse effects were reported in both groups. The improvement in total SF-12 score on Day 30 for the SR vs SC group was 82.79% vs 80.04% respectively. In addition, our Naive Bayes classifier based on the leave-one-out model achieved the maximum accuracy (68.30%) for the classification of both groups of uPID.

**Conclusion:** We concluded that the SR group is cost-effective, safer, and efficacious for curing uPID. Proposed alternative treatment (test drug) could be a substitute of standard drug used for Female genital tract infections.

## 1 Introduction

Uncomplicated pelvic inflammatory disease (uPID) is a serious and common infection of the female genital organs in reproductive-aged women (Reekie et al., 2018; Lu et al., 2020; Qayyum et al., 2023; Sultana et al., 2023). Uterine inflammation/female reproductive tract inflammation can be translated as *Waram al-Rahim*. The clinical features of reproductive tract infections including pelvic inflammatory disease are analogous to *Waram al-Rahim* clinical features. In the absence of a definite diagnosis, most of the participants usually and mistakenly consider a psychogenic cause for their pain, thus leading to dissatisfaction. Various microorganisms such as *Mycoplasma genitalium, Neisseria gonorrhoeae*, and *Chlamydia trachomatis* are related to bacterial infections and play a significant role in uPID (Filho, 2018; Reekie et al., 2018).

**FIGURE 1:**
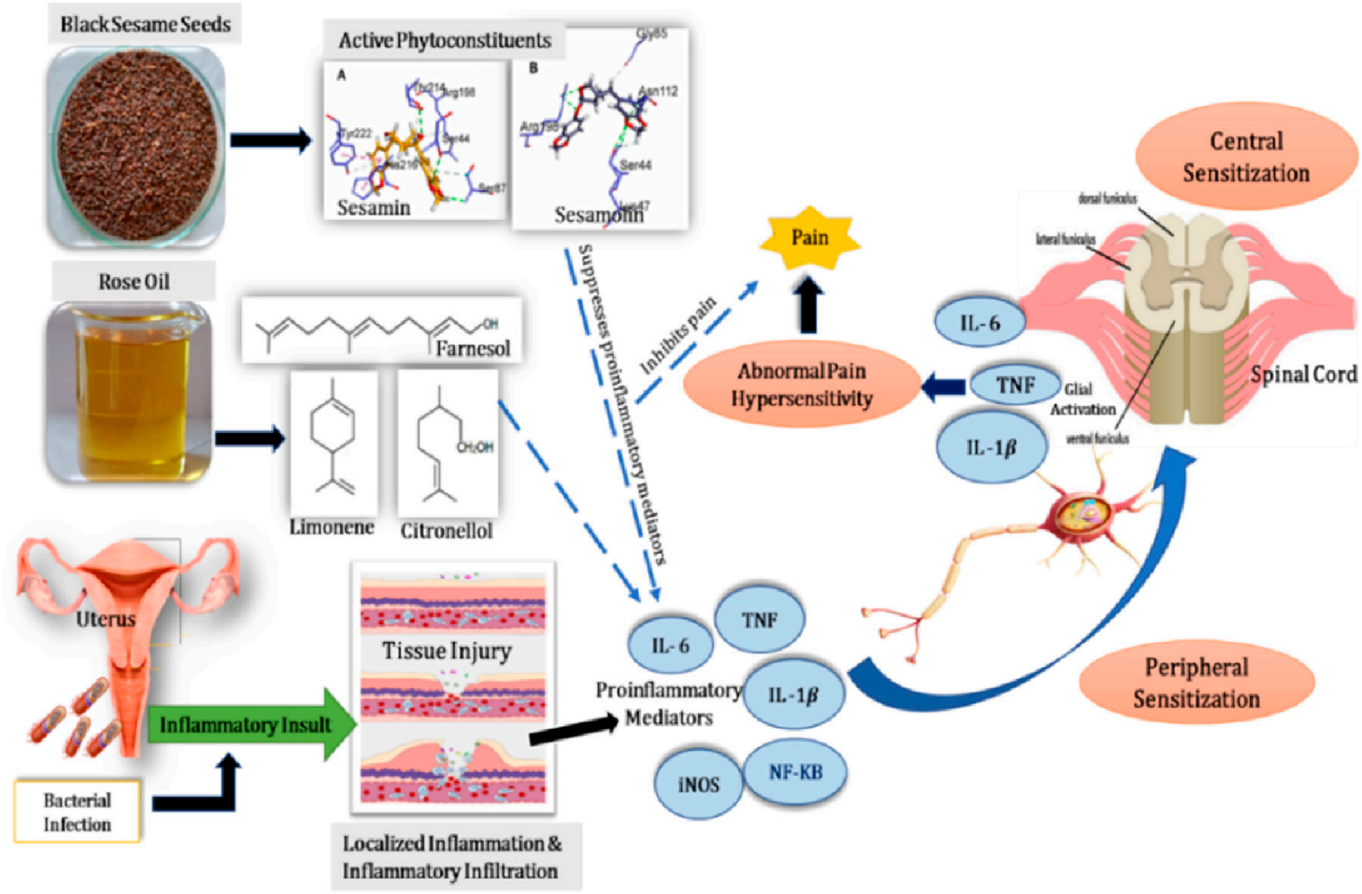
Mechanism of inflammation, anti-inflammatory, and analgesic activities of sesame and rose oil.

As per conventional medicine, PID is upper genital tract and surrounding structures infection-induced inflammation in women that results from a lower genital tract infection (Dewitt and Hegazi, 2014; Savaris et al., 2019; Jennings and Krywko, 2020; Xu and Gray-Owen, 2021). The pathogenesis of pelvic pain/inflammation and central nervous system (CNS) related changes that accompany it are still unknown. The International Association for the Study of Pain (IASP) describes pain as a product of higher brain center processing that is a painful emotional and sensory experience related to definite or potential damage to the tissue which applies to date. Noxious stimuli and direct activation of nociceptors in nerve endings in the tissues lead to tissue damage or inflammation, which causes nociceptive pain. The pro-inflammatory markers interleukin (IL)–1, IL-6, inducible nitric oxide synthase (iNOS), nuclear factor kappa B (NF-kB), and tumor necrotizing factor (TNF) have a direct effect on central sympathetic activity and nociceptive nerve fibers (Figure 1). Numerous interconnections exist between the anatomical structures of the pelvis and the nervous system, as well as in the various pelvic organs. The mechanisms of perception of pain continue to have interest and have attracted many physicians and scientists, even though they are still largely unknown. The two important phenomena that have been proposed to be related to chronic pelvic pain are hypothalamic–pituitary–adrenal (HPA) axis dysregulation and central sensitization (Asiri et al., 2019). Chronic pelvic discomfort has been related to emotional, behavioral, negative cognitive, and sexual problems. Pelvic pain is a common problem that has a detrimental influence on health-related quality of life (HRQoL). It is assumed that central disturbances in pain processing and viscerosensory signals play a role in pelvic pain/inflammation (Asiri et al., 2019). Sensory information from pelvic organs is carried by hypogastric plexus and pudendal nerves and then painful sensations are conveyed from the pelvis to the brain through thoracolumbar and sacral dorsal root ganglia cells. The pain afferent pathways for sensory input are suppressed or augmented in these brain circuits and spinal cord descending pathways. Hence, the intensification of nociceptive signals is perhaps responsible for widespread pain in the CNS (Origoni et al., 2014; Asiri et al., 2019).

In conventional medicine, symptoms of PID are vaginal discharge, fever, cervical motion tenderness, lower abdominal pain, dyspareunia, adnexal and uterine tenderness, or abnormal uterine bleeding (Saif et al., 2019; Savaris et al., 2019). A clinical examination with positive endocervical sample results for microbes supports the diagnosis of PID. Yet, negative microbiological findings do not rule out this diagnosis (Brun et al., 2020). Participants with chronic pelvic pain are more likely to have comorbid psychiatric problems. Furthermore, these are linked to higher pain severity and lower quality of life. The overlap between chronic pain and psychological illnesses is complicated, but it appears that environmental, genetic, inflammatory, and neurological variables all contribute to increased vulnerability to both of these conditions (Gartstein. et al., 2016). Intervention in uPID aims to relieve the pain and systemic symptoms caused by infection, achieve a clinical/microbiological cure, and prevent the spread of infection. It further prevents complications and minimizes adverse effects (Ross, 2013). Antibiotics used in modern medicine have certain adverse effects on the human body. Natural, botanical drugs or traditional herbal medicine is presently being studied with great curiosity and hope. Hence, complementary and alternative treatments for uPID are required which are safe, easily available, and efficacious in treating the condition.

Various Unani botanical drugs are useful in uPID such as *Solanum nigrum* L. (*Mako*)*, Althaea officinalis* L. (*Tukhme Khatmi*)*, Berberis aristate* DC. (*Raswat Zard*)*, Achillea millefolium* L. (*Brinjasif*)*, Cichorium intybus* L. (*Kasni*), *Trigonella foenum-graecum* L. (*Methi*)*, Rosa damascena* Mill (*Gule Surkh*), *Plantago ovata* Forssk (*Isapghol*)*,* and *Linum usitatissimum* L. (*Katan*) (Ghani, 2001; Kabir-al-Din, 2007). Pharmacologically, these botanical drugs are proven to have antibacterial, analgesic, antipyretic, and anti-inflammatory properties (Khare, 2007). Various previous clinical studies also have proven the efficacy of Unani and botanical drugs (Agarwal, 2014; Mohebitabar et al., 2017). However to date, in Unani medicine, in all types of uterine inflammation, *R. damascena* Mill oil (rose oil) with *Sesamum indicum* L. (black sesame seeds) in the form of a pessary (*Hamul*) has not been validated (Mohammad, 2006).

*Sesamum indicum* L. (Sesame) belongs to the family Pedaliaceae also known as *S. orientale* L. (Anonymous, 1997; Rivera et al., 2014) and *Tukhm Kunjad Siyah* in Unani medicine (Anonymous, 1997). Sesame seeds are a significant source of protein, oil, minerals, and carbohydrates for human nutrition (Zhou et al., 2016). The bioactive component sesamin, which is derived from sesame, has been discovered to shield the liver from oxidative damage. Sesame seeds have natural antibacterial effects against common skin pathogens such as *Staphylococcus* and *Streptococcus* spp. They were also found to have antifungal, antiviral, and anti-inflammatory properties. Flavonoids and other phenolic plant metabolites found in sesame seeds have been shown in prior research to have antioxidant properties (Dravie et al., 2020). Black sesame seeds possess anti-inflammatory (*Muhallil-i-Awram*), analgesic (*Musakkin-i-Alam*), and astringent properties*,* as per Unani texts (Kabir-al-Din, 2007; Tariq, 2010; Khan and Muhammad, 2018). Pharmacologically, ethanol extracts of sesame seeds were tested for anti-microbial activity against a variety of microorganisms (*P. mirabilis*, *Erwinia coli*, *P. aeruginosa, Staphylococcus aureus,* and *C. albicans*) (Linn, 2021). Experimental studies have also confirmed the anti-inflammatory (Henriques Monteiro et al., 2014; Wu et al., 2019), analgesic (Henriques Monteiro et al., 2014; Linn, 2021), and antioxidant (Linn, 2021) properties of black sesame seeds.

Rose oil in Unani medicine is known as *Roghan Gul.* It is made using four different processes from garden rose petals. (Mahboubi, 2019). Rose oil is recommended not only for inhalation and topical application but also for oral administration at physiologically applicable doses (Mileva et al., 2021). It possesses anti-inflammatory (*Muhallil-i-Awram*), analgesic (*Musakkin-i-Alam*), and astringent properties*,* as per Unani texts (Ghani, 2001). Rose oil’s antibacterial activity has been proven against *P. aeruginosa, E. coli*, *B. subtilis, S. aureus, Erwinia carotovora,* and *C. violaceum* strains in an experiment (Ulusoy et al., 2009). Therefore, this study sought to determine the therapeutic efficacy and safety of *S. indicum* Linn seeds with *R. damascena* Mill Oil in uPID with standard control. Additionally, we analyzed the data with machine learning.

The main contributions of this study were to design a novel drug from the combination of *S. indicum* L. powder with *R. damascena* Mill oil for the treatment of uPID. This novel drug would be efficacious in treating uPID as it would have antimicrobial, analgesic, anti-inflammatory, and antioxidant activities as proven in research studies. We explored the relationship between antimicrobial, antioxidant, anti-inflammatory, and immunomodulatory activities in uPID with *in-vitro* and *in-vivo* studies. Furthermore, we designed a heatmap for the experimental features and an artificial intelligence (AI)-enabled automatic classification model.

## 2 Materials and methods

### 2.1 Experimental design and ethical statement

The experimental design was a double-blind, randomized, standard-controlled, double-dummy trial. The study was conducted in the Dept. of Obstetrics and Gynaecology, National Institute of Unani Medicine, Bangalore. This trial received Ethics Committees approval from our Institute (NIUM/IEC/2019-20/012/ANQ/04). This study was registered at the clinical trial registry of India (CTRI), ICMR with ID No CTRI/2021/01/030182 before initiating the clinical trial**.** This study was performed as per the “*Declaration of Helsinki*” and “*GCP”* guidelines of the Ministry of AYUSH, India. We have taken consent from each randomized patient included in the study.

### 2.2 Participant selection

For the inclusion criteria, married women from 18 to 45 years diagnosed with clinical features of uPID as per ACOG guidelines (lower abdominal pain, adnexal and cervical motion tenderness, abnormal vaginal discharge, pelvic discomfort, Low Backache, dyspareunia, and dysuria) were included. In the present study participants with other features of uPID (mild/subclinical PID) were also included.

For the exclusion criteria, **c**omplicated cases of PID including pelvic abscess and/or any condition likely to require any surgical intervention within 24 h were excluded. Furthermore, participants with any malignancy and systemic disease, on OCPs (Oral contraceptive pills), IUCD (Intrauterine contraceptive device), pregnant, lactating women, delivery, and abortion within the last 3 months were excluded (Judlin et al., 2010).

### 2.3 Data collection

Detailed demographic and clinical parameters were enquired about during the interview to diagnose uPID. Socioeconomic status was determined by Kuppuswamy’s scale. At each visit, a visual analog scale (VAS) for lower abdominal pain (LAP) and Low Backache (LBA) and a modified McCormack tenderness pain scale (McPS) were used to assess pelvic tenderness. *Before the vaginal examination,* participants *were asked to empty their bladder. A local examination for excoriation, edema, and erythema was done. During the gynecological examination vaginal discharge’s quantity, color, consistency, and odor were noted. A Pap smear and vaginal wet mount were also performed.* All the clinical findings were recorded by the researcher. The participants were requested to avoid other medicines during the trial period. They were also advised of the barrier method precaution during intercourse. In addition, each participant was instructed regarding the use of vaginal pessary and oral medicines and to maintain perineal hygiene.

Hematological and biochemical markers such as complete hemogram, *erythrocyte sedimentation rate,* urine analysis, C reactive protein, and random blood sugar were performed for the exclusion of general diseases. To exclude sexually transmitted diseases, serological markers such as Venereal Disease Research Laboratory (VDRL), human immunodeficiency virus (HIV), and Hepatitis surface antigen (HBsAg) were also performed. *To diagnose cervical malignancy and bacterial vaginosis, a Pap smear was performed. To diagnose PID and pelvic pathology, ultrasonography of the pelvis was performed*. Safety evaluation included physical examination findings and laboratory biochemical markers.

For safety, hematological and biochemical markers including hemogram, hepatic function tests (aspartate aminotransferase (AST), alanine aminotransferase (ALT), alkaline phosphatase (ALP)), and renal function tests (blood urea, and serum creatinine) on day 0 and day 15 were done. *The participants were instructed* to report any adverse effects related/not related to the treatment. At each follow-up, all findings were entered into the case record forms.

### 2.4 Assessment tools

The abnormal vaginal discharge score was calculated by visual analog scale score (VAS). *A sterile spatula was introduced for the collection of discharge from the vagina (posterior fornix and sidewall) for the vaginal wet mount test. A small amount of vaginal discharge was spread over a glass slide with a few drops of normal saline and covered with a transparent glass slip* to check the presence of white blood cells per high field in the vaginal wet mount smear under the microscope (Crossman, 2006; Jaiyeoba et al., 2011).

*For the diagnosis of BV, three out of four of Amsel’s criteria were considered significant* (Mohammadzadeh et al., 2015)*.* The VAS score for pain intensity is the most commonly used validated tool (Savaris et al., 2007). *The scoring of each symptom scale was graded as absent (0), mild (1–3), moderate (4–6), Severe (7–9), and very severe (10) (Boonstra et al., 2008)*. *T*he health-related quality of life was measured by a Short Form (SF)-12 scale (Shou et al., 2016).

### 2.5 Intervention

#### 2.5.1 Selection of plant materials and identification of SR group

After reviewing the literature, various Unani formulations and single drugs having properties of antiseptic (Dafi’-i-Ta’affun), anti-inflammatory (Muhallil-i-Waram), astringent (Qabid) and tonic (Muqawwi) effective in uPID were explored (Ghani, 2001; Kabir-al-Din, 2007). Pessary of black sesame powder with rose oil was selected for the treatment of uPID in the form of pessary as they are useful in uPID (Waram-al-Rahim) (Kabiral-Din, 2006). It has antimicrobial, anti-inflammatory, antioxidant, and analgesic properties (Mohebitabar et al., 2017).

The details of trial plant materials were presented as per ConPhyMP guidelines (Heinrich et al., 2022). All trial-related medications were provided by our institute pharmacy. One week before the start of the research, the Institute pharmacist directly purchased the plant materials from the neighborhood market. The drug was sent for identification and confirmation at the Foundation for Revitalisation of Local Health Traditions (FRLHT), University of Trans-Disciplinary Health Sciences and Technology, Bengaluru, India. The pharmacognosist identified the plant materials as R. damascena Mill flower and S. indicum L. seeds belonging to the family Rosaceae and Pedaliaceae respectively. For future reference, the specimen has been reposited in our Institute (Voucher specimen numbers were 105/UQ/Res/2021/1 and 105/UQ/Res/2021/2).

*Sesamum indicum* L. is a species of plant that is widely grown around the world, primarily in tropical and subtropical areas, particularly in Burma, India, China, and Sudan. Around the world, sesame seeds are preferred to be added as a seasoning on food products (Zhou et al., 2016). India has the greatest sesame cultivation area and produces 27.9% of the world’s sesame (Wu et al., 2019). It is an erect annual herb that grows 0.3–0.9 m high. White, black, and yellow sesame seeds are the three types of seeds, while white and black sesame seeds are the most popular (Wang et al., 2018). Macroscopically, black sesame seeds (*Tukhm Kunjud Siyah*) are black ovoid, laterally compressed, and very small, about 3–4 mm long, 2 mm broad, and 1 mm thick. One end is broad and tapers towards the hilum (Anonymous, 1997). The seed coat’s texture may be either smooth or rough. The seeds are delicately punctate and have four enigmatic longitudinal ridges at the borders of the flat faces. The hilum is at the pointed end, and the raphe extends as a line from the center of one flat face to the broader end (Wang et al., 2018). The epidermis of the seed consists of thin-walled palisades, and the anticlinal walls are more or less curved. The epidermis’ cells measure 50–95 mm in length and 18–30 mm in width (Anonymous, 1997; Wang et al., 2018). Based on the powder analysis, it is oily, heterogeneous, and black with a sweet taste and distinctive oily scent. An abundance of big epidermal cells, palisade cells, and isodiametric cells of the parenchyma of various sizes, each densely loaded with the protein bodies, were visible upon microscopic analysis of the powder (Anonymous, 1992).

Sesame seeds are significant sources of protein, oil, minerals, and carbohydrates for human nutrition (Zhou et al., 2016). A study measured the free, bound, and total phenolic contents of black sesame seeds. Total phenolic contents were measured by a modified Folin-Ciocalteu colorimetric method. Black sesame seed displayed a maximum of 4.99 ± 0.47 of free and 2.33 ± 0.36 g GAE kg^−1^ of bound phenolic compounds. The total phenolic contents in black sesame varied from 4.54 to 7.32 gm GAE kg^−1^ (Zhou et al., 2016). A study analyzed the lignans including sesamol, sesamin, and sesamolin in free and bound extracts of black sesame seeds by HPLC using methanol/water (75: 25 v/v) with 0.1% formic acid in water as mobile phase. Overall, higher levels of lignans (>89%) were present in free phenolic extracts. The black sesame showed 82.83–251.91 mg kg^-1^ of lignans in free phenolic extracts. The highest content of sesamol, sesamolin, and sesamin in the free phenolic extract was 187.25 ± 10.56 mg kg^-1^, 25.12 ± 0.95 mg kg^-1^, and 39.55 ± 0.38 mg kg^-1^ respectively (Zhou et al., 2016). Nadeem et al. (Nadeem et al., 2014) found that the total phenolics extracted were 1.72 gm GAE kg^−1^ from sesame cake. Shahidi et al. (Shahidi et al., 2006) confirmed that the total phenolic contents of black sesame contain 29.9 ± 0.6 gm catechin equivalents kg^−1^ in crude ethanolic extract.

*Rosa damascena* Mill [family: Rosaceae] is commonly known as *Gul-e-Surkh* in Urdu and *Warde Ahmar* in Arabic (Anonymous, 2006). It originates in Iran, and since the seventh century A.D., human beings have been extracting essential oils from its blossoms (Mahboubi, 2016). Around the world, roses have been extensively grown in regions with moderate weather. Roses are grown in China, Japan, Korea, and India (Kashmir, Bihar, Uttar Pradesh, and Punjab States) in addition to Europe, North America, and Northwest Africa (Morocco). More than 90% of the world’s rose oil is produced in Bulgaria, Turkey, and Iran, though *R. damascena* oil from Bulgaria is the highest-quality oil available. It is a 2.5 m tall perennial shrub that has numerous robust branches. They have 5 to 60 pale pink to pinkish red corolla petals (Mileva et al., 2021). The cell of the upper epidermis in the petal is rectangular to squarish or radially elongated, thick-walled with yellowish-brown contents, and coated with thick cuticles on the outside surface. Mesophyll is distinguished by compact, polygonal to oval, thick-walled parenchymatous cells with slightly uneven walls (Anonymous, 2006). Supplementary Table S1A summarizes the taxonomy (Rosa damascena Mill, 2023; Sesamum indicum, 2023), and phytochemical parameters of black sesame seeds (Anonymous, 1997) and rose flowers (Anonymous, 2006; Fathima and Murthy, 2019).

Rose oil in Unani medicine is known as *Roghan Gul.* Rose oil is made using four different processes from garden rose petals (Mahboubi, 2019). Supplementary Table S1B summarizes the phytochemical parameters of rose oil prepared by traditional methods (Anonymous, 1986). Thin layer chromatography of pet. ether extract of rose oil using mobile phase pet. ether: diethyl ether: acetic acid (90: 10:1) and stationary phase sprayed with 10% H2 SO4, exhibit three spots at *Rf.* values of 0.09, 0.30, and 0.48. When pet. ether: diethyl ethers: acetic acid was used in an 80:20:1 ratio and the stationary phase was treated with 10% H2SO4, the chromatogram showed three spots at *Rf.* values of 0.23, 0.37, and 0.68 (Anonymous, 1986).

#### 2.5.2 Preparation of the trial medicine and dispensing

*Black* sesame seeds were finely powdered with the powder-making machine (Hammer mill), sieved with mesh number 80, and separately kept in small airtight packets.

Each participant received 14 sachets, each containing 5 g of powder. The sachets were packed in a sealed aluminum pouch according to the calculated dose of 5 g powder each.

The rose oil (*Roghan Gul*) was prepared by the traditional method. An equal quantity of fresh rose petals was separated from rosehips and mixed in boiling sesame oil at 170°C till the watery part was dried (10 min) and then filtered (Kabir-al-Din, 2007; Khan and Muhammad, 2018; Mahboubi, 2019). Each participant received 14 sachets, each containing 10 mL of rose oil. These sachets were packed in a sealed aluminum pouch.

#### 2.5.3 Administration, and dosage

Participants in the SR group (n = 30) received 14 days course of black sesame powder (5 g) mixed with rose oil (10 mL) cotton pessary (*Hamul*) per vaginum at bedtime once daily plus two placebo capsules (500mg, edible cellulose powder) orally. Each participant received a unique batch of 28 capsules that were given out in an opaque aluminum pouch. Participants in the SC group (n = 30) received 14 days course of standard, doxycycline 100 mg twice and metronidazole 400 mg thrice orally plus a placebo (pessary of cellulose and distilled water) per vaginum at bedtime.

#### 2.5.4 Training of the participants

Fourteen sterilized cotton tampons were given to each participant, each separately wrapped in an aluminum pouch. Each participant received instructions on how to fill a measuring cup with 10 mL of oil, add one sachet of powder, and thoroughly mix the ingredients. They were then instructed to insert the tampon, place it deep inside the vagina at night, and then remove it in the morning.

#### 2.5.5 Compliance

Compliance with the treatment was evaluated by asking the participants to return the empty packets at the end of the protocol duration. This number would then be compared with the total number of units received by the participants.

### 2.6 Outcomes

The main outcome included a change in VAS for LAP, LBA, and McPS for abdominal-pelvic tenderness. The primary efficacy endpoints for the study were a clinical cure, for both VAS and McPs scales, and a 65% or higher reduction in a score at day 15 and day 30 compared to baseline (Savaris et al., 2007).

The secondary outcome included a change in the SF-12 HRQoL questionnaire, WBC cell on saline microscopy of vaginal discharge in vaginal wet mount test, and safety profile. For the secondary efficacy endpoint, improvement of quality of life was defined as a 70% or greater improvement in the score at day 30 when compared to baseline for total component summary, physical component, mental component summary, and each domain of the SF-12 questionnaire. The clinical cure was defined as WBC <10/HPF in a wet mount test. The percentage reduction (PR) score of the showVAS score for pain and McPS was also calculated at each follow-up from the baseline as shown in Eq. 1:

$$\mathrm{PR}\;\left(\%\right)=\frac{mean\;score\;at\;follow\;up\;-\;mean\;score\;at\;day\;0}{Mean\;score\;at\;day\;0}*100$$ (1)

### 2.7 Randomization, blinding allocation, and matching

Computer-generated randomization and allocation of 1:1 for a simple random technique were performed and an open list of random with a single block was computed. The randomization number was concealed from the first investigator and pathologist until the trial and control medicines were assigned to each participant. The participant *and pathologist* who reported vaginal wet mount tests for the presence of white blood cell count were blinded in the study. Pessary and oral medicines were dispensed in non-transparent sealed sachets in the SR and SC groups to match both groups. The participants of both groups were called at different periods so that participants could not interact and be able to know the treatment they received. As the drugs were packed in air-tight sealed sachets so there was no obvious rose oil aroma noted.

### 2.8 Sample size

Based on an outcome of clinical cure with the difference in two groups, 28%, a level of significance of 5% with a statistical power of 90%, and a sample size of 60 (30 in each group) was adequate for a two-group randomized study including an attrition rate of 10% (Savaris et al., 2007).

### 2.9 Statistical analysis

Data were imputed in Microsoft Excel and the statistical software SPSS version 28 was used to perform data analysis. A two-sided *p*-value, Alpha error was 0.05, 95 percent confidence interval, and statistical power of 90% were imputed. The chi-square test/Fisher’s exact test was used to compare the socio-demographic variables between the group and the independent *t*-test for quantitative variables. Intergroup comparison for VAS score LAP, LBA, and tenderness was performed by using the Mann-Whitney *U* test and intragroup comparison used the Friedman test. All efficacy factors were examined using data from all randomized participants who had received at least one dose of the SR group or SC drug (first day after visit 1), according to the principles of intent-to-treat to impute missing data, the last observation was carried forward (McCoy, 2017). *p* > 0.05 was considered not significant.

### 2.10 Machine learning techniques

The machine learning technique is a type of artificial intelligence (AI) technique (Akhtar et al., 2020; 2023; Iqbal et al., 2022; Parveen et al., 2023). We used AdaBoost (AB), Naive Bayes (NB), and Decision Tree (DT) machine learning classifiers on the SR (sesame and rose oil) and the SC (Doxycycline and Metronidazole) groups related to uPID.

#### 2.10.1 AdaBoost

AB is one type of ensemble classifier. It is the combination of different classifiers that improves classification performance. Each classifier is trained using a simple set of training samples. In addition, each piece has weight, and the consequences of all models are familiar iteratively (Chola et al., 2021; Pal et al., 2022). Standard equations of AB are described in (2)-(4) given below:

$$Y\left(x\right)=sign\left[\sum_{m=1}^{n}\alpha_{m}y_{m}\left(x\right)\right]$$ (2)

$$\alpha_{m}=0.5\ln\left(\frac{\left(1-e\right)}{e}\right)$$ (3)

$$D_{m+1}\left(i\right)=\frac{D_{m}\left(i\right)e^{\left(-\alpha_{m}y_{i}y_{m}\left(x_{i}\right)\right)}}{Z_{m}}$$ (4)

#### 2.10.2 Naive bayes

The NB classifier was constructed on the Bayes theorem with free expectations between predictors (Xu, 2018). Bayes theorem offers to calculate the posterior probability P_PID_ (tp) from the prior probability of target P_PID_ (t), the prior probability of predictor P_PID_ (p), and the probability of predictor given target P_PID_ (pt). The NB classifier accepts the effect of the value of a prior probability of predictor on a given target (Bin Heyat et al., 2021; Heyat et al., 2021; AlShorman et al., 2022). The standard equation of the NB classifier is described in Eq. 5:

$$N_{B\;\left(PID\right)}=P_{PID\;}\left(\text{tp}\right)\frac{P_{PID}\left(p_{t}\right)P_{PID}\left(t\right)}{P_{PID}\left(p\right)}$$ (5)

#### 2.10.3 Decision tree

The DT Classifier is a supervised machine-learning technique, where information is constantly separately applicable to different constant values. It has two separate stages’ *viz.* classification tree and reversion tree. Here, in this article, the classifier is a few dual parameters of the random moment in trees that are divided into small subparts which must be in the range of five (Heyat et al., 2019; Lai et al., 2019; Belal Bin Heyat et al., 2022; Bin Heyat et al., 2022; Tripathi et al., 2022). The main benefits of this classifier are that computation time is fast and de-noising is embedded in it. The standard Equations 6, 7 are given below:

$$E\left(H_{t}\right)=\sum_{j}P_{j}H_{j}$$ (6)

$$R_{t}=H-E\left(H_{t}\right)$$ (7)

Where *H*_*t*_ is the regular indecision after execution of test t, *P*_*j*_ is the chance that the examination has a j outcome, and *R*_*t*_ is the regular lessening of doubt attained by examination t.

## 3 Results

**FIGURE 2:**
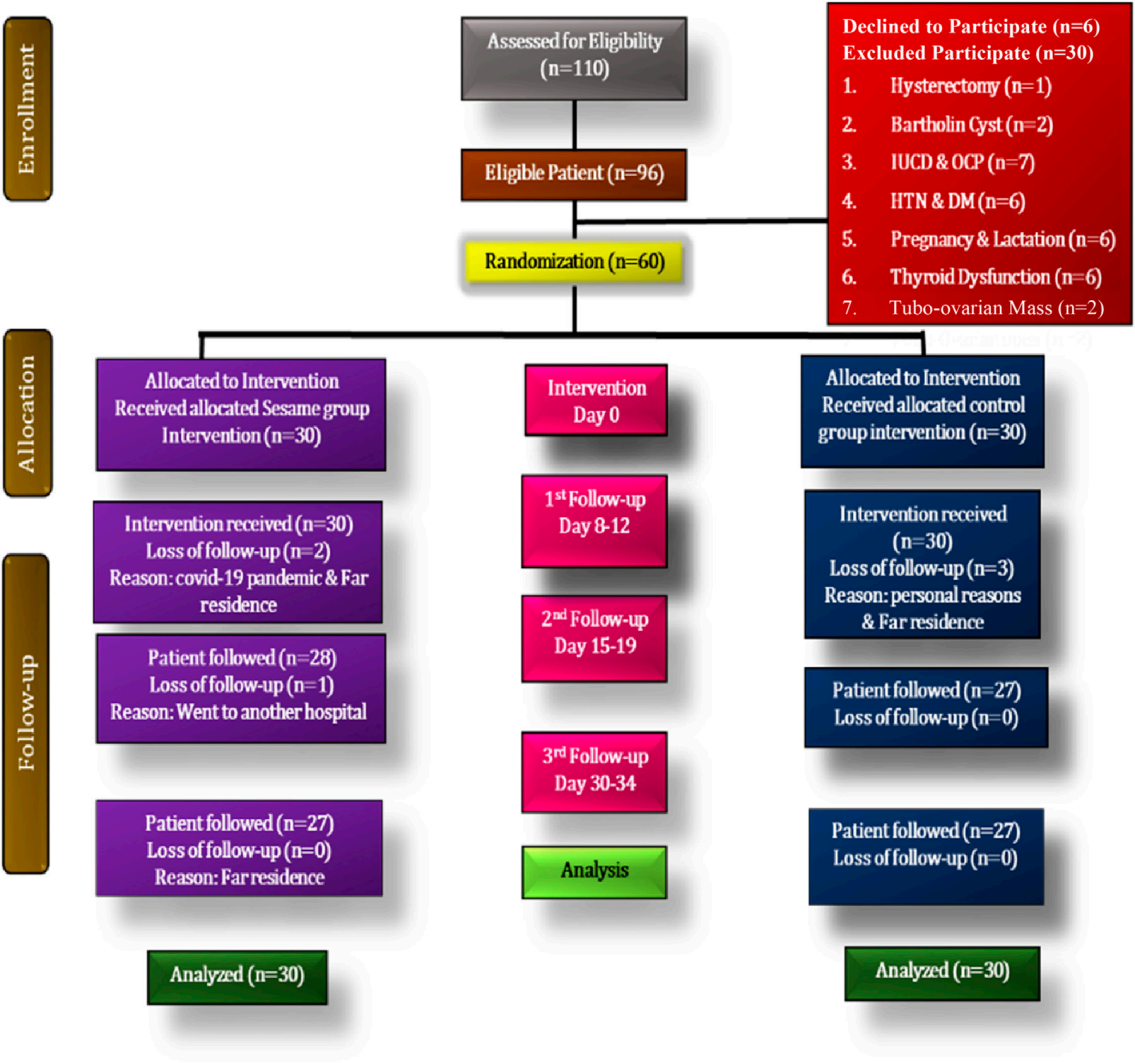
Flow chart as per consort statement in the proposed study.

Participants who fulfilled the study criteria were randomly allocated either to the SR or SC intervention group. A total number of 110 participants were evaluated for PID as per ACOG guidelines. A total of 96 participants were diagnosed with PID of which 6 declined to take part in the study. Thirty participants were excluded for different reasons. Then, the remaining 60 participants were randomized for the study. The first patient was enrolled on 25 February 2021 and the last follow-up of the last patient was completed on 30 January 2022 from the outpatient of our Institute. Three patients were lost to follow-up in the SR group and 3 patients were lost to follow-up in the standard group. However, 30 patients in each group were included as the analysis was carried out as per the intent to treat principle (McCoy, 2017) (Figure 2).

### 3.1 Sociodemographic variables, routine investigations, and pap smear

Statistical tests demonstrated insignificant differences in the baseline parameters between the groups (*p* > 0.05), showing homogeneity at baseline (Table 1). At baseline, serological markers (HIV, VDRL, and HbsAg) were negative for all participants.

**TABLE 1:** Baseline sociodemographic parameters of the participants in both groups.

Variables	SR Group (n=30)	SC Group (n=30)	Total (*n*=60)	p-value
**Age (year)**	31.13±7.12	30.93±6.30		0.90^a^
≤ 20	2 (6.66)	2 (6.66)	4 (6.66)	
21-30	12 (40)	12 (40)	24 (40)	0.98^b^
31-40	12 (40)	13 (43.3)	25 (41.6)	
41-50	4 (13.3)	3 (9.67)	7 (11.6)	
Religion				
Muslim (1)	26 (86.6)	27 (90)	53 (88.3)	1.00^c^
Hindu (2)	4 (13.3)	3 (10)	7 (11.6)	
Habitat				
Rural (1)	2 (6.66)	4 (13.3)	6 (10)	0.67^c^
Urban (2)	28 (93.3)	26 (86.6)	54 (90)	
Diet				
Vegetarian (1)	2 (6.66)	3 (10)	5 (8.3)	1.00^c^
Non-vegetarian (2)	28 (93.3)	27 (90)	55 (91.6)	
**BMI (Kg/m** ^**2**^ **)**	24.95±5.32	25.45±4.98		0.41^d^
<18.5	2(6.66)	2(6.66)	4 (6.66)	
18.5-24.9	14(46.66)	11(36.66)	25(41.66)	
25-29.9	11(36.6)	10(33.33)	21(35)	0.57^b^
>30	3 (10)	7(23.33)	10(16.66)	
**Socioeconomic parameters**				0.64^c^
Upper	0	0	0	
Upper middle	2(6.66)	2(6.66)	4(6.66)	
Lower middle	12(40)	10(33.33)	22(36.66)	
Upper lower	16(53.33)	16(53.33)	32(53.33)	
Lower	0	2(6.66)	2(3.33)	

^a^ Unpaired *t*-test.

^b^ Chi-square test.

^c^ Fisher Exact test.

^d^ Mann Whitney *U* test; data presented in mean ± SD, or No (%).

On day 0, in the SR group 20 (66.6%), 6 (20%), and 4 (13.3%) participants had an inflammatory smear, bacterial vaginosis, and normal smear respectively, whereas, in the SC group, 22 (73.3%), 7(23.3%), and 1 (3.33%) patient had inflammatory, bacterial vaginosis, and normal smear respectively. On day 15, 14 (46.6%), 2 (6.66%), and 14 (46.6%) in the SR group, whereas 18 (60%), 3 (10%), and 9 (30%) participants in the SC intervention, had inflammatory, bacterial vaginosis, and normal smear respectively.

### 3.2 Primary outcome results

#### 3.2.1 VAS score for LAP

The pre- and post-intervention comparison was statistically significant at each follow-up in both SR and SC groups (*p* < 0.001). The percentage reduction of VAS score was 82.85% and 81.48% in the SR and SC groups respectively at day 15 from baseline. The percentage reduction of VAS score was 89.04% and 87.96% in the SR and SC groups respectively at day 30 from baseline.

#### 3.2.2 VAS score for LBA

The intragroup comparison was statistically significant at each follow-up in both SR and SC groups (*p* < 0.001). The percentage reduction of VAS score was 61.71% and 50.53% in the SR and SC groups respectively at day 15 from baseline. The percentage reduction of VAS score was 68.31% and 53% in the SR and SC groups respectively at day 30 from baseline.

#### 3.2.3 McPS

**FIGURE 3:**
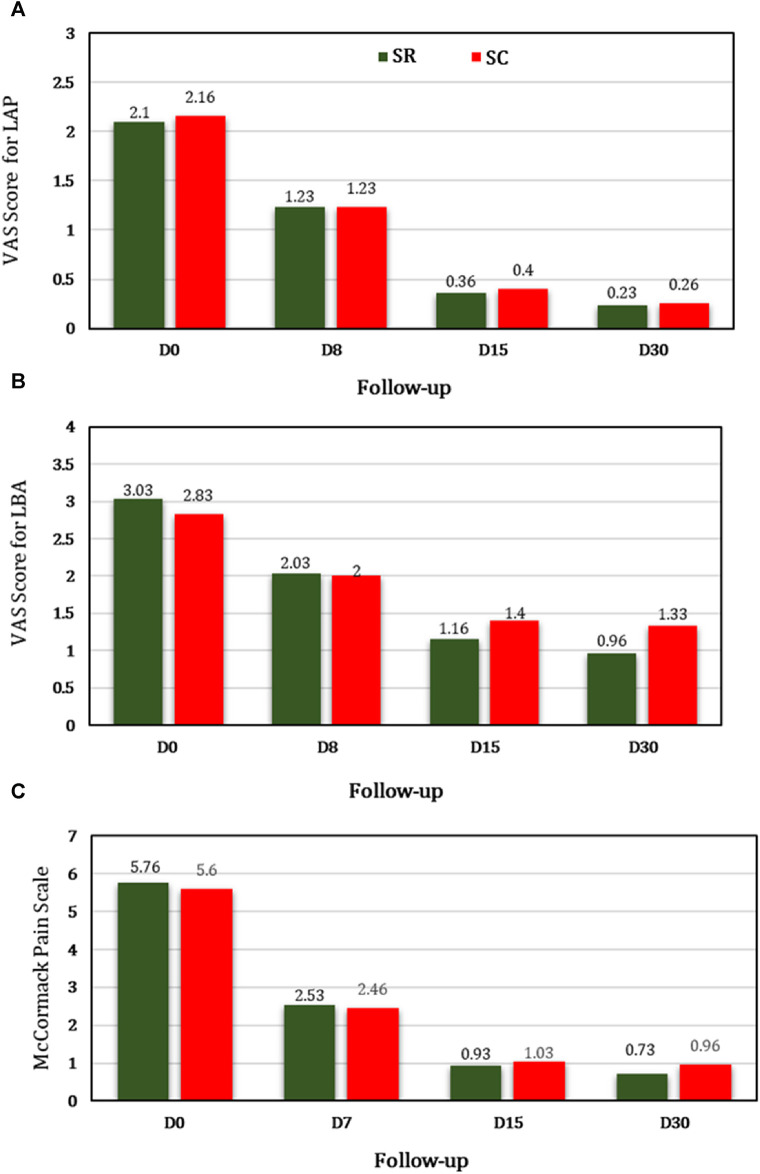
Comparative analysis between SR and SC groups for the primary outcomes **(A)** LAP, **(B)** LBA, and **(C)** McPS.

The intragroup comparison was statistically significant at each follow-up in the SR and SC groups (*p* < 0.001). The percentage reduction was 83.85% and 81.60% in the SR and SC groups respectively at day 15 from baseline. The percentage reduction was 87.32% and 82.85% in the SR and SC group respectively at day 30 from baseline (Table 2; Figure 3).

**TABLE 2:** Primary outcomes (VAS and McPS) in both (SR and SC) groups.

Follow-up	SR group (n = 30)	95% CI (LL-ul)	SC group (n = 30)	95% CI (LL-ul)	*p-*value
Visual analogue scale (VAS) for lower abdominal pain (LAP)					
**D0**	2.1 ± 0.48	1.92-2.27	2.16 ± 0.53	1.96-2.36	0.82
**D8**	1.23 ± 0.56^***^	1.02-1.44	1.23 ± 0.56^***^	1.02-1.44	0.99
**D15**	0.36 ± 0.76^***^	0.08-0.65	0.4 ± 0.77^***^	0.11-0.68	0.84
**D30**	0.23 ± 0.72^***^	−0.03-0.50	0.26 ± 0.73^***^	−0.009-0.54	0.84
**%PR at D15**	82.85	-	81.48	-	-
**%PR at D30**	89.04	-	87.96	-	-
VAS for Low Backache (LBA)					
**D0**	3.03 ± 0.49	2.85-3.21	2.83 ± 0.64	2.59-3.07	0.18
**D8**	2.03 ± 0.55^***^	1.82-2.24	2 ± 0.58^***^	1.78-2.21	0.82
**D15**	1.16 ± 0.83^***^	0.85-1.47	1.4 ± 0.67^***^	1.14-1.65	0.23
**D30**	0.96 ± 1.03^***^	0.58-1.35	1.33 ± 0.75^***^	1.05-1.61	0.12
**%PR at D15**	61.71	-	50.53	-	-
**%PR at D30**	68.31	-	53	-	-
Modified McCormack Pain Scale (McPS) for Pelvic Tenderness					
**D0**	5.76 ± 1.67	5.41-6.39	5.6 ± 1.03	5.21-5.98	0.75
**D8**	2.53 ± 1.30^***^	2.04-3.02	2.46 ± 1.27^***^	1.98-2.94	0.85
**D15**	0.93 ± 1.81^***^	0.25-1.61	1.03 ± 1.86^***^	0.33-1.73	0.81
**D30**	0.73 ± 1.78^***^	0.06-1.39	0.96 ± 1.84^***^	0.27-1.65	0.41
**%PR at D15**	83.85	-	81.60	-	-
**%PR at D30**	87.32	-	82.85	-	-

***p< 0.001 considered extremely significant from baseline at each follow-up; Test used: Mann Whitney *U* test and Friedman test; %PR: percentage pain reduction.

### 3.3 Findings of the secondary outcomes

#### 3.3.1 HRQoL

**FIGURE 4:**
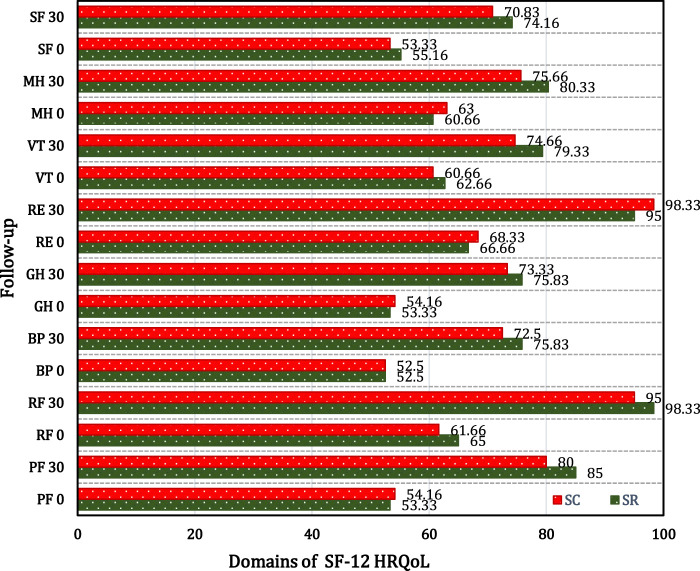
SF-12 health survey domain comparison in SR and SC groups.

**FIGURE 5:**
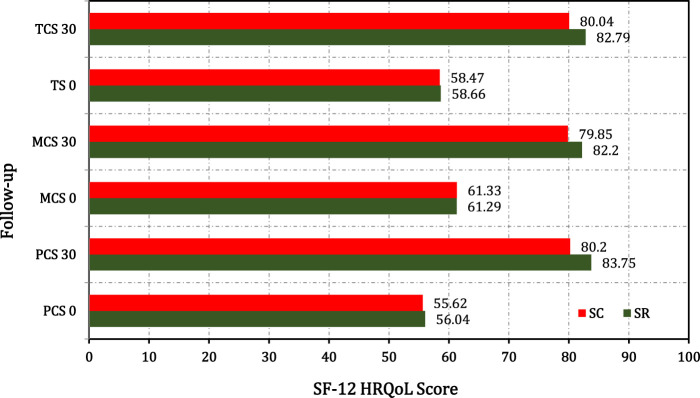
Comparative analysis between SR and SC groups for the secondary outcome measure such as total component score, physical and mental component sub scores of SF-12 HRQoL.

The intragroup comparison of both groups at day 30 from baseline showed extremely significant improvement (*p* < 0.0001) in all parameters of the SF-12 score. The mean percentage improvement of the total SF-12 score was 82.79% and 80.04% in the SR and SC groups respectively at 30 days from baseline (Figures 4, 5).

#### 3.3.2 WBC/HPF in vaginal wet mount test

On day 0, pus cells were present >10 in the wet mount test in all the participants of the SR and SC group (n = 60, 100%). On day 15, a significant reduction in pus cells from baseline in both groups (*p* < 0.001) was noted. On day 15, 26 (86.6%) and 23 (76.6%) participants had less than 10 pus cells in the SR and SC groups respectively with an insignificant difference (*p* > 0.05). The intragroup comparison from baseline to day 15 was statistically significant (*p* < 0.001) (Table 3).

**TABLE 3:** Vaginal wet mount test and hematological and biochemical biomarker parameter assessment of both groups.

Investigations	SR Group (n=30)	SC Group (n=30)	*p*-value
WBC/HPF in Vaginal Wet Mount Test			
BT	31.2±25.37	29.7±25.23	0.80^a^
AT	5.7±8.09	8.1±634	0.12^a^
*p*-value	<0.0001^a^	<0.0001^a^	
Hb% (gm %)			
BT	11.60±1.34	11.88±1.45	0.44^d^
AT	11.57±1.21	11.87±1.31	0.36^d^
*p-value*	0.80^c^	0.30^b^	
ESR			
BT	20.4±14.47	18.1±9.37	0.46^d^
AT	17.76±10.69	18.03±9.53	0.91^d^
*p-value*	0.24^c^	0.97^c^	
TLC (cells/cumm)			
BT	7057.03±1860.7	7896.6±1839.9	0.19^e^
AT	7206.66±1534.0	7943.33±1539.7	0.06^d^
*p-value*	0.78^b^	0.86^c^	
Neutrophils			
BT	58.96±7.11	58.86±7.68	0.95^d^
AT	57.45±6.84	58.46±7.74	0.5^d^
*p*-value	0.32^c^	0.78^c^	
Lymphocytes			
BT	33.36±6.26	34.06±6.66	0.48^d^
AT	34.33±5.87	34.53±6.68	0.90^d^
*p*-value	0.39^c^	0.71^c^	
S. Creatinine (mg/dl)			
BT	0.86±0.13	0.81±0.12	0.16^d^
AT	0.86±0.13	0.83±0.13	0.92^d^
*p*-value	0.45^b^	0.70^b^	
Blood Urea (mg/dl)			
BT	20.23±6.21	22.73±7.41	0.17^e^
AT	20.9±5.2	21.56±6.96	0.76^e^
*p*-value	0.49^b^	0.34^b^	
Alanine Aminotransferase (IU/L)			
BT	30.2±11.98	27.5±6.71	0.28^d^
AT	30.9±10.32	30.8±8.67	0.96^d^
*p*-value	0.73^c^	0.11^c^	
Aspartate Transaminase (IU/L)			
BT	23.7±10.09	22.5±5.78	0.58^d^
AT	22.6±7.14	24.0±6.47	0.42^d^
*p*-value	0.53^c^	0.14^c^	
Alkaline Phosphatase (IU/L)			
BT	94.6±17.9	96.03±21.1	0.77^d^
AT	92.9±11.5	92.3±19.8	0.88^d^
*p*-value	0.57^c^	0.17^c^	

^a^ Fisher exact test.

^b^Unpaired *t*-test.

^d^Wilcoxon Matched paired test.

^c^ Paired *t*-test.

^e^ Mann Whitney *U* test.

#### 3.3.3 Safety parameters

The intergroup comparison showed that both groups were homogenous at day 0 and day 15 with no statistical difference (Table 3) showing that hematological (hemogram) and biochemical parameter (ALT, AST ALP, blood urea, and serum creatinine) assessment before and after treatment had no changes. This shows that SR and SC group treatments were harmless to the liver and kidney, proving no toxicity of these medicines on the body functions. Furthermore, no patients in either group complained of any side effects or serious adverse events.

### 3.4 Associated symptoms of uPID

#### 3.4.1 Abnormal vaginal discharge

On day 0, all participants *had abnormal vaginal discharge* in the SR and SC groups whereas, on day 30, 25 (83.4%) and 12 (40%) participants in the SR and SC groups respectively had no abnormal vaginal discharge (*p* = 0.007).

#### 3.4.2 Dyspareunia

On day 0, a total of 17 (56.6%) participants in the SR group and 13 (43.3%) participants in the SC group had complaints of dyspareunia, whereas, on day 30, 14 (46.6%) participants in the SR group and 12 (40%) participants in the SC group had no dyspareunia (*p* = 0.0002).

#### 3.4.3 Dysuria

On day 0, 4 participants in the SR group and SC groups had complaints of dysuria, whereas, on day 30, no patient had dysuria in the SR group and only 1 patient in the SC group had dysuria with no statistical difference (*p* = 0.32 was statistically insignificant).

### 3.5 Machine learning classification results

We classified the SR and SC (Doxycycline and Metronidazole) groups in terms of area under the curve (AUC), precision, F1, accuracy, recall, specificity, and log loss. Additionally, we used the AB, NB, and DT machine-learning classifiers with cross-validation (CV) 5-fold, 10-fold, and leave-one-out models. The mathematical expression for the precision, F1, accuracy, recall, and specificity (Ullah et al., 2022b; 2022a; Sultana et al., 2022a; Ayalew et al., 2022; Nawabi et al., 2022; Said et al., 2022; Benifa et al., 2023; Pal et al., 2023; Qadri et al., 2023) are shown in Equations 8-12 below:

$$precision=\left(\frac{TP}{FP+TP}\right)$$ (8)

$$F1=\frac{\left(2\times recall\times precision\right)}{\left(recall+precision\right)}$$ (9)

$$accuracy=\left(\frac{TP+TN}{TP+FP+TN+FN}\right)$$ (10)

$$recall=\left(\frac{TP}{TP+FN}\right)$$ (11)

$$specificity=\left(\frac{TN}{\left(FP+TN\right)}\right)$$ (12)

Where true positives are TP, false positives are FP, true negatives are TN, and false negatives are FN.

**FIGURE 6:**
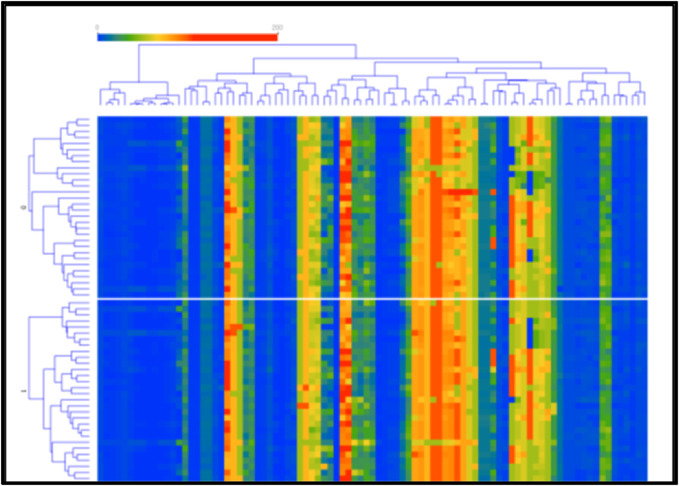
Heat map of the SR and SC groups for the uPID.

We calculated a heat map of the SR and SC group information related to uPID, as shown in Figure 6. It showed the association between the experimental and demographic features for both uPID groups. In the CV 10-fold model, the NB classifier achieved maximum accuracy in terms of precision (61.80%), F1 (61.60%), accuracy (61.70%), recall (61.70%), and specificity (61.70%). In the CV 5-fold model, the AB classifier accomplished maximum accuracy concerning precision (63.40%), F1 (63.33%), accuracy (63.33%), recall (63.33%), and specificity (63.33%). In addition, the NB classifier for the leave-one-out model achieved maximum accuracy in terms of precision (68.50%), F1 (68.30%), accuracy (68.30%), recall (68.30%), and specificity (68.30%). While, the average precision, F1, accuracy, recall, and specificity were found to be 62.10%, 61.90%, 62.00%, 62.00%, and 62.00%, respectively. Nevertheless, our NB leave-one-out model is more appropriate for the classification of the SR and SC group in uPID, as shown in Table 4 and Figure 6.

**TABLE 4:** Classification results between SR and SC groups related to uPID using machine learning classifiers.

Classifier	Model	AUC	Precision	F1	Accuracy	Recall	Specificity	Log loss
AB	CV-10	0.600	0.600	0.600	0.600	0.600	0.600	13.816
NB		0.719	0.618	0.616	**0.617**	0.617	0.617	1.465
DT		0.649	0.617	0.617	0.617	0.617	0.617	10.501
AB	CV-5	0.633	0.634	0.633	**0.633**	0.633	0.633	12.664
NB		0.654	0.620	0.614	0.617	0.617	0.617	1.721
DT		0.677	0.584	0.582	0.583	0.583	0.583	7.202
AB	**Leave-one-out**	0.567	0.569	0.562	0.567	0.567	0.567	14.967
**NB**		**0.740**	**0.685**	**0.683**	**0.683**	**0.683**	**0.683**	**1.298**
DT		0.692	0.667	0.667	0.667	0.667	0.667	9.837
**Mean**		0.659	0.621	0.619	0.620	0.620	0.620	8.163
**±SD**		0.051	0.034	0.035	0.034	0.034	0.034	5.176
**Variance**		0.002	0.001	0.001	0.001	0.001	0.001	26.799

## 4 Discussion

This study showed the clinical cure for LAP was 82.85% and 89.04% on day 15, and 81.48% and 87.96% on day 30, in the SR and SC interventions, respectively. The clinical cure for McPS was 83.85% and 87.32% on day 15, and 81.60% and 82.85% on day 30 in the SR and SC groups, respectively. The total SF-12 score improvement was 82.79% and 80.04% in the SR and SC groups respectively. The hematological (hemogram) and biochemical biomarker parameter (ALT, AST ALP, blood urea, and serum creatinine) assessments before and after treatment had no changes. This shows that the SR and SC group interventions were safe on the body functions. Furthermore, no patients in either group complained of any side effects or serious adverse events. On day 15, 86.6% and 76.6% of participants had less than 10 pus cells in the SR and SC groups respectively with an insignificant difference (*p* > 0.05). The intragroup comparison from baseline to day 15 was statistically significant (*p* < 0.001).

*Two studies have shown that v*aginal neutrophils with more than 10/HPF in vaginal wet mount have a sensitivity of 78% and a specificity of 39% (Jaiyeoba et al., 2011). Few investigators have reported the absence of vaginal white blood cells had a 95% negative predictive value for PID. The absence of neutrophils in the vaginal wet mount smear suggests an alternative diagnosis to PID (Crossman, 2006; Jaiyeoba et al., 2011). *Globally, VAS is widely used to measure the intensity of pain. It is a valid, reliable, and interval scale* (Boonstra et al., 2008). Saif et al. have found a highly statistically significant reduction of pain and resolution of clinical features in the first group of patients (Saif et al., 2019). A study has found a reduction in clinical features determined by VAS scores (Zahid and Rehman, 2016). Sayed et al. found clinical responses for VAS in 90% and 95% of the test and control groups and pelvic tenderness and vaginal discharge were cured in 90% and 95% of patients respectively in both groups (Sayed and Shameen, 2016). Another study has shown an improvement in 21 patients out of 30 for subjective and objective parameters (*p* < 0.0001) (Parween et al., 2017). Qayyum et al. found a significant improvement in VAS score for LAP, LBA, and McPS scores before and after treatment, consistent with our study (Qayyum et al., 2023).

A previous study reported that abnormal vaginal discharge is the most common presenting complaint (Lata et al., 2019). Saif et al. have found a significant improvement in vaginal discharge before and after treatment, which is consistent with this study (Saif et al., 2019). A study conducted to examine the effect of *L. usitatissimum* L. (*Tukhme Katan*) and *P. ovata* Forssk (*Isapghol*) in *Waram al-Rahim* showed that the test drug improved abnormal vaginal discharge in 28 out of 30 patients (Qayyum et al., 2023). In this study, on day 30, 46.6% of participants in the SR group and 40% of participants in the SC group had no dyspareunia. Previous studies also reported that dyspareunia is a presenting symptom of PID (Parween et al., 2017; Al-kuran et al., 2021). On day 30, no participant had dysuria in the SR group and one participant in the SC group had dysuria. In addition, we noted that participants with bacterial vaginosis (BV) also responded to the SR treatment. Another study confirmed the usefulness of *Pistacia integerrima* J. L. Stewart ex Brandis in BV (Baig et al., 2022).

### 4.1 Black sesame and rose oil usefulness in pelvic inflammatory disease

Various pharmacological properties have been found, including the anti-inflammatory, analgesic, antioxidant, immunomodulatory, antimicrobial, anticancer, hepatoprotective, nephroprotective, and antiproliferative activity of black sesame seeds (Majdalawieh et al., 2020; Linn, 2021). *Rosa damascena* has shown versatile pharmacological activities *viz.*, antimicrobial, anti-inflammatory, analgesic, antimutagenic, antioxidants, anticancer, free-radical scavenger, and antidepressant activity (Akram et al., 2020).

### 4.2 Anti-microbial properties

**FIGURE 7:**
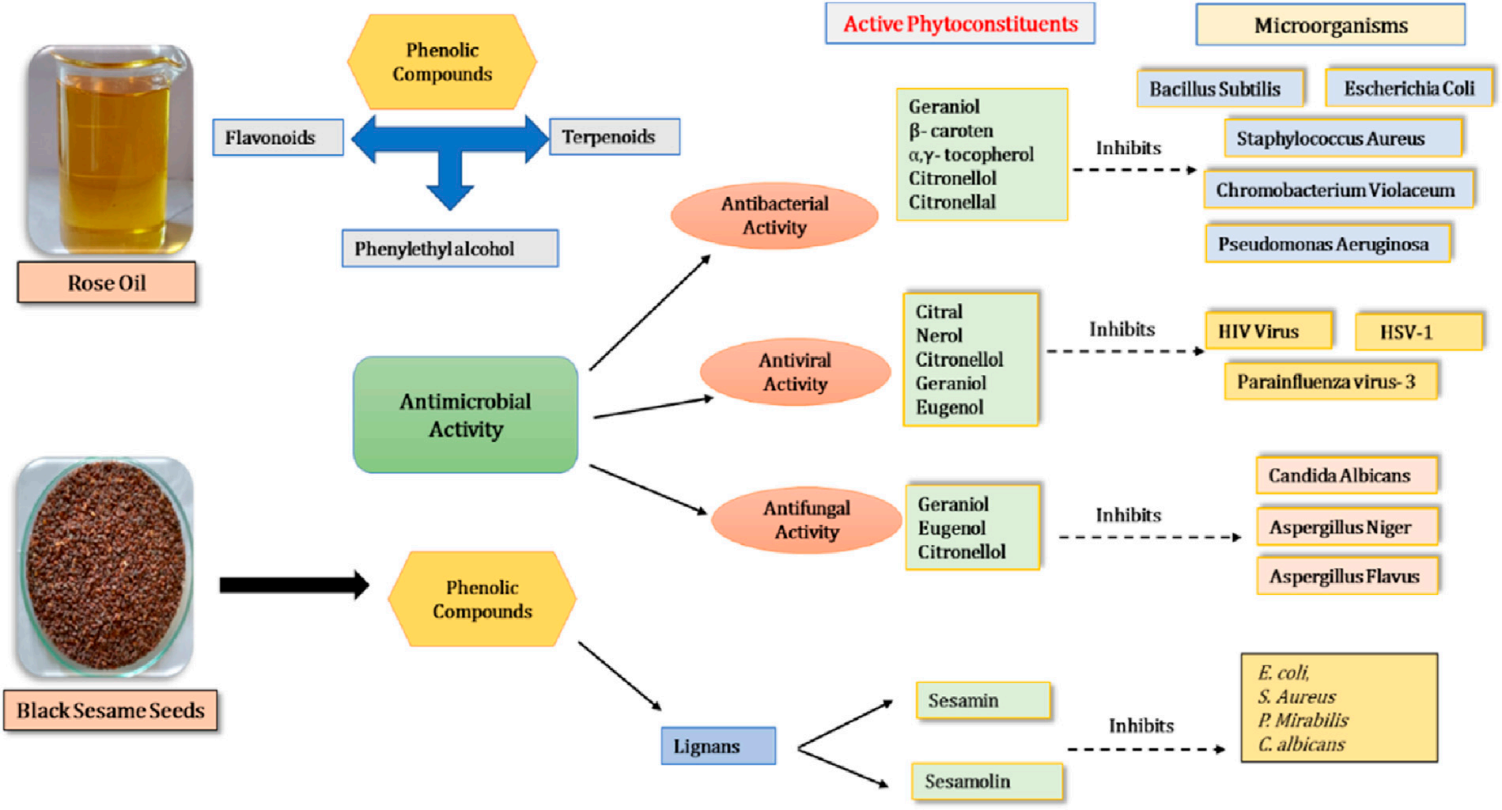
Antimicrobial activity of black sesame and rose oil.

The ethanolic extracts and hot and cold water of the sesame seeds showed anti-microbial activity against tested microorganisms. The presence of lignans (phenolic) and other metabolites confirmed significant antibacterial and antioxidant activities in the methanolic extract of *S. indicum* (Linn, 2021). The researchers reported that the rose oil demonstrated antibacterial potential against Gram-positive and Gram-negative bacteria (*Pseudomonas aeruginosa, B. subtilis, E. coli*, *C. violaceum, S. aureus,* and *E. carotovora* strains). The rose essential oil’s antimicrobial activity is perhaps due to the presence of monoterpenes and their lipophilic nature, which causes loss of its high permeability for protons and larger ions as they disrupt the microbial cytoplasmic membrane and compromise its function. Citronellol, geraniol, and nerol, the main components of rose oil, demonstrated considerable antibacterial activity against some microorganisms (Ulusoy et al., 2009). Therefore, these ingredients may operate as a mediator for the antibacterial activity of rose oil (Boskabady et al., 2011). Further, citronellal, citral, nerol, geraniol, citronellol, and eugenol are common components that showed antiviral activity (Mileva et al., 2021) (Figure 7).

### 4.3 Anti-inflammatory and analgesic properties

Sesame seeds have anti-inflammatory (Ghani, 2001; Kabir-al-Din, 2007) properties which can be the reason for the reduction of pus cells in the wet mount test. Pelvic discomfort is a major problem for both patients and physicians. It deteriorates the QoL of the patients and also affects their social relationships. Additionally, pelvic pain is annoying to patients as well as to doctors, who attempt to recognize the pathologic reason to rationalize the clinical condition.

Pelvic pain disorders can be caused by (i) nociceptive, (ii) inflammatory, (iii) neuropathic, (iv) psychogenic, (v) mixed, and (vi) idiopathic biological processes. Inflammatory pain is caused by the body’s response to tissue damage and the subsequent inflammatory process, which can activate “silent nociceptors”. These nociceptors do not ordinarily reply to thermal or mechanical stimuli. Inflammation and the inflammatory response are the fundamental cause of pain of any type. Inflammatory mediators are released and sensory nerve fibers are stimulated when soft tissue or nerves are injured. Cytokines, growth factors, neuropeptides, and neurotransmitters are biological mediators of inflammation. At the peripheral terminals of these fibers, the release of inflammatory neuropeptides is triggered by the antidromic firing of these sensory nerves. These neuropeptides have the potential to promote vasodilation, enhance vascular permeability, recruit T helper cells, and activate sensory nerve fibers in the surrounding area. This condition is known as neurogenic inflammation. Furthermore, local tissue inflammation causes secondary hyperalgesia as well as central sensitization. Hence, this causes diffuse muscle pain, joint pain, fever, lethargy, spasm, and anorexia-like syndrome (Omoigui, 2009). An *in vitro* study proved that in response to *N. gonorrhoea,* prostaglandin E2 is produced from dendritic cells and leukotriene B4 from neutrophils which are the lipid mediators important for inflammatory hyper nociception. In women with acute PID, peritoneal fluid showed elevated levels of both mediators, hence targeting these responses may help to provide pain relief (Xu and Gray-Owen, 2021). Inflammatory response and inflammation are the sources of all pain. Hence, inflammation in PID can also cause pain.

Black sesame has an anti-inflammatory effect by inhibiting COX2 activity or PGE2 synthesis in non-animal and animal models. Other studies have revealed that some of these constituents suppress pro-inflammatory cytokines such as IL-1β, TNF-α, IL-6, and NF-kB in a rat model or a cell culture model (Afroz et al., 2019). Sesame lignans hamper the dissemination of inflammatory mediators and inflammatory cytokines, hence they exhibit the extenuation of inflammatory-related pathways (Wu et al., 2019). Further, sesame also has antinociceptive activity and a study proved that ethanolic extract of *S. indicum* (500 mg/kg) in mice produced a significant analgesic effect similar to ibuprofen (50 mg/kg) (Linn, 2021). Studies reported that bioactive sesame molecules act on opioid agents (specific central antinociceptive), and exert their analgesic activity through supraspinal and spinal receptors (Henriques Monteiro et al., 2014). Hence, we hypothesize that because of the aforementioned mechanism, sesame seeds are able to inhibit the inflammatory biochemical mediator and thereby reduce inflammation and pain. Furthermore, sesame seeds also have immunomodulatory and antioxidant properties. Monteiro et al. reported that Sesamin is one of the active ingredients in sesame oil, which supports the product’s antinociceptive and anti-inflammatory characteristics (Henriques Monteiro et al., 2014).

In a study, the hydroalcoholic extract of *R. damascena* showed analgesic and anti-inflammatory potential in mice. Previous studies reported that rose oil has anti-inflammatory and anti-trichomonas properties and, hence is probably effective in cervicitis. The local application of rose oil is useful for uterine diseases especially cervicitis, wounds, and burns (Saghafi et al., 2021). Another study reported that the severity of primary dysmenorrhea was reduced by a massage with rose oil (Shahr et al., 2015) and relieved pregnancy-related LBA without any significant side effects (Akram et al., 2020). The beneficial effects of rose oil with other essential oils for reducing menstrual pain and bleeding were also reported (Mohebitabar et al., 2017). Polyphenols and flavonoids are present in *R. damascena* Mill. oil have been shown to have a considerable influence on cyclooxygenase, showing anti-inflammatory effects (Mileva et al., 2021). Furthermore, the presence of flavonoids, kaempferol, and quercetin in *R. damascena* has an analgesic effect (Akram et al., 2020). According to a study, plant components that are not soluble in water may be the cause of the analgesic effect that has been seen in hydroalcoholic and ethanolic extracts. As a result, it has been hypothesized that the water-insoluble plant metabolites quercetin and kaempferol may be accountable for this effect (Boskabady et al., 2011). Some major plant metabolites of rose oil include limonene, cis-rose oxide, farnesol, and citronellol which have shown anti-inflammatory activity in animal studies. Limonene inhibits pro-inflammatory mediators and decreases NF-κB activity, ROS production, and eosinophil migration. Cis-rose oxide suppresses IL-1ß production and leukocyte migration**.** Farnesol was also found to have anticancer activity and citronellol also has antispasmodic, antibacterial, and antifungal activities (Mileva et al., 2021). Further other pharmacological activities in rose oil are astringent and refrigerant properties (Ghani, 2001). Hence, black sesame and rose oil were able to reduce abdominal pain, backache, pelvic tenderness, and vaginal discharge.

### 4.4 Antioxidant and immunomodulatory activities

**FIGURE 8:**
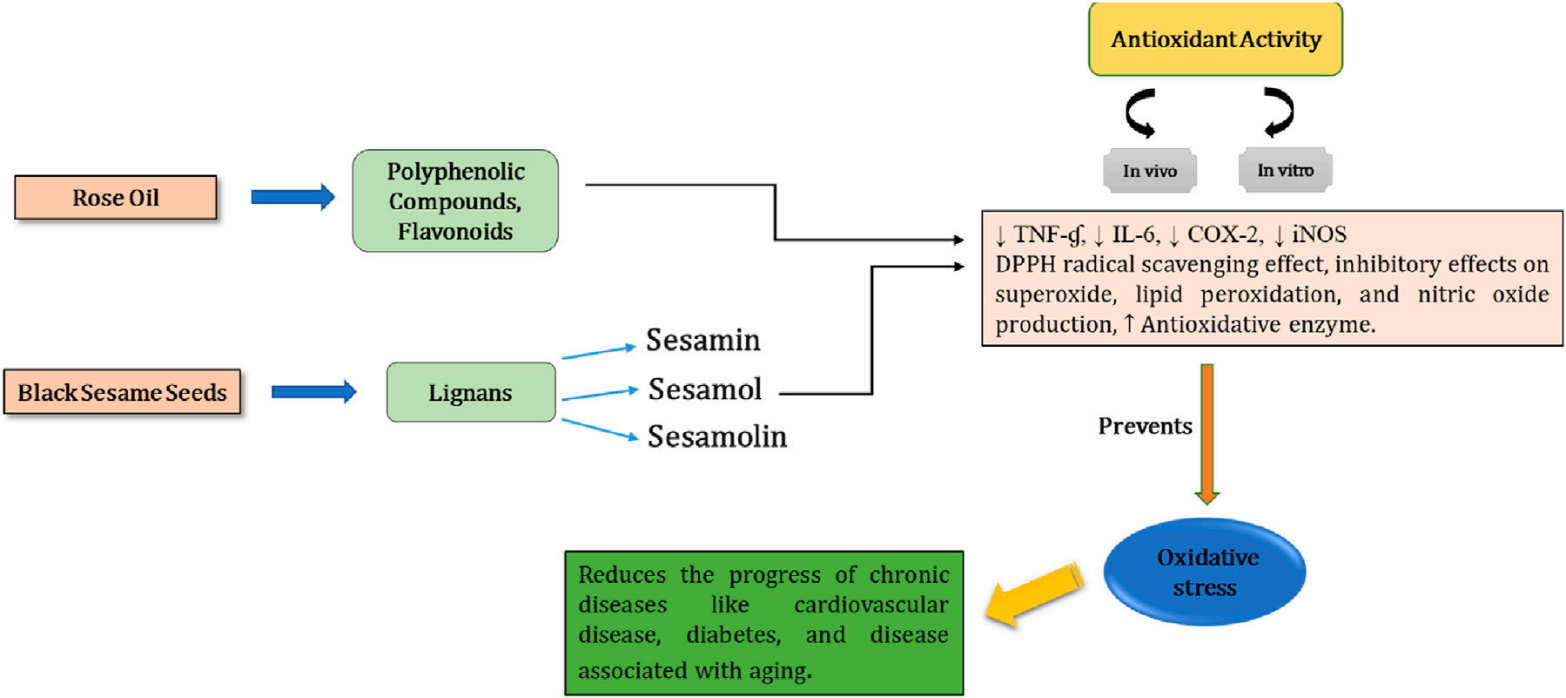
Antioxidant activity of black sesame and rose oil.

The improvement in quality of life may be due to the presence of tonic (*Musammin-i-Badan*)*,* aphrodisiac (*Muqawwi-i-Bah*)*,* and nutritive (*Mugharri*) properties in black sesame seeds (Ghani, 2001)*.* Black sesame seeds also exhibited galactagogue, hepatoprotective, and diuretic properties. The presence of lignans helps in increasing hepatic fatty acid oxidation enzymes and because of their phytosterol content, black sesame seeds help to strengthen the body’s immune system (Linn, 2021). Oxidative stress progresses when the body’s antioxidant system is exhausted. The main cause of various chronic disorders is an increase in the free radical concentration inside cells including reproductive-related problems. Antioxidant phytochemicals reduce the progress of many chronic diseases by neutralizing free radicals (Sultana et al., 2022b; Fazmiya et al., 2024) (Figure 8). Zahra et al. suggested that the effects of ROS involve biological roles as weapons in the arsenal of the immune system (Zahra et al., 2021).

Sesamol, sesamol, sesamin, sesamolin, and gammatocopherol are natural antioxidants and sesamol is the main component that has been shown to have antioxidant properties in non-animal and animal studies through the reduction of superoxide, lipid peroxidation, and nitric oxide production and increased antioxidative enzyme. It also provides high oxidative stability and prevents oxidation followed by free radical mechanisms (Henriques Monteiro et al., 2014; Afroz et al., 2019). Zhou et al. reported that black sesame seeds’ antioxidant activities were associated with total phenolics and flavonoids (Zhou et al., 2016).

Rosaceae family plants are enriched with plant metabolites and have numerous biological activities. The study showed that *R. damascena* has high antioxidant potential (Ansari et al., 2017; Mileva et al., 2021). Furthermore, *R. damascena* mill oil has demonstrated remarkable antioxidant activities in chemical, biological, and experimental models in mice for oxidative stress. Lipid peroxidation revealed that pre-treatment with antioxidants Trolox, vitamin C, and *R. damascena* mill essential oil reduced oxidative stress markers. The combined effects of damask rose oil and L-DOPA were remarkably comparable to those of Trolox and vitamin C (Mileva et al., 2021). Antioxidant activity of flavanol glycosides of ethanolic extract including kaempferol- 3-O-arabinoside, kaempferol-3-O-rhamnoside, and quercetin-3-O-glucoside have been reported (Ansari et al., 2017). Phenolic chemicals, which are antioxidants present in nature, play an essential part in determining antioxidant potential. They claimed that *R. damascena*, an oil-bearing rose, has the highest antioxidant capacities. A study claimed that the high concentration of phenolic chemicals, particularly free gallic acid, is primarily responsible for the rose petal’s high level of radical scavenging action.

### 4.5 Hepatoprotective and nephroprotective studies of black sesame seed and rose oil

Studies have also confirmed the hepatoprotective (Linn, 2021), immuno-modulator (Linn, 2021), neuroprotective (Henriques Monteiro et al., 2014), cardioprotective (Majdalawieh et al., 2020), and nephroprotective (Wang et al., 2018) properties of black sesame seeds. The *S. indicum* extract was used at the dose range of 1–100 μg/mL for all experiments because this dose range was shown to have no toxicity (Majdalawieh et al., 2020). Nwachukwu et al. in their study determined the hepatoprotective activity of *S. indicum* in rats (Nwachukwu et al., 2011). Azab et al. (Azab, 2014) in their study included 32 adult male albino mice in four groups, 8 in each, to investigate the hepatoprotective activity. In albino mice, sesame oil exhibited potential hepatoprotective activity against lead acetate-induced hepatotoxicity (Azab, 2014). Monteiro et al. (Henriques Monteiro et al., 2014) in their study showed that the sesame oil was not toxic to the animal at doses received after 48 h of treatment. After treatment with oil, the acute toxicity test on mice exhibited that none of the animals died in 48 h. The authors concluded the safety of sesame oil in therapeutic applications (Henriques Monteiro et al., 2014). Hsu et al. investigated the effect of sesame oil on nephrotoxicity caused by the collaborative action of aminoglycoside and iodinated contrast in Sprague-Dawley rats (Hsu et al., 2011). The rise of serum blood urea nitrogen and creatinine levels was significantly prevented by sesame oil. Hori et al. (Hori et al., 2011) reported that sesamin and episesamin have no genotoxic activity.

A toxicity study of *R. damascena* showed that the medication may be hepatotoxic at extraordinarily high doses (Ansari et al., 2017). After incubation for 1 h, *R. damascena* essential oil (1%) killed human lymphocytes *in vitro* and the same effect was observed with 0.1% after 24 h (Mileva et al., 2021). The authors concluded that the hepatoprotective action of *R. damascena* extracts was interrelated to its antioxidative activity (Alam et al., 2008). In rats, *R. damascena,* oral LD50, and rose absolute was >5 g/kg whereas, in rabbits dermal, *R. damascena* and LD50 was >2.5 g/kg (Lis-Balchin, 2006). In albino rabbits, the nephroprotective activity of aqueous extract of 250 and 500 mg/kg of *R. damascena* has been evaluated on gentamicin (80 mg/kg) induced toxicity by using silymarin (200 mg/kg). The results supported significant improvement in renal cortical histopathology (Khaliq et al., 2015).

### 4.6 Strengths, limitations, and future recommendations of the present study

This was a first-of-its-kind study in clinical intervention in which the efficacy of pessary of black sesame with rose oil to reduce the VAS for LAP and LBA, and McPS for abdominopelvic tenderness and improvement of HRQoL was investigated. Further, it was a double-dummy, double-blind, randomized standard-controlled study, the sample size was calculated, intent-to-treat analysis was performed and patient compliance was good with a 10% dropout. The SR group was safe without any adverse effects. No clear evidence of adverse events related to antibiotics, leading to discontinuation of therapy, was observed.

The limitation of the present study was the smaller sample size due to the COVID-19 policy. The second limitation was specific diagnostic tests such as cervical swab culture and nucleic acid amplification test (NAAT) were not performed to diagnose the microbiological cure rate as we lacked the infrastructure and time. Hence, we recommend the aforementioned specific diagnostic tests for future studies. This trial was to authenticate and validate the Unani pharmacopeia medicine for its safety and efficacy. We also suggest that standardization, dosage form development, and quantitative analysis of the metabolites responsible for their pharmacological activities including antimicrobial and anti-inflammatory potentials are required along with stability appraisal of the finished product. In the future, research studies are required to prove the pharmacokinetics, pharmacodynamics, and efficacy of the SR intervention on antioxidant and anti-inflammatory biomarkers to prove their activities.

## 5 Conclusion

Our findings indicate that cotton pessary of black sesame with rose oil has a therapeutic similar effect to the standard drug in the treatment of uPID. Besides our findings also showed that SR was safe and also enhanced the HRQoL of women with uPID. The research drugs were efficacious in curing uPID as they possess antimicrobial, anti-inflammatory, analgesic, and antioxidant activities. Further, these activities were because of the presence of sesamin and sesamolin in sesame and phenolic plant metabolites present in rose oil. Besides, our NB Classifier easily classifies the both (SR and SC) groups. In addition, we would apply our classification methods to the other female infectious diseases.

## Data Availability

The raw data supporting the conclusion of this article will be made available by the authors, without undue reservation.
