# Supplementary material for: Efficacy and classification of Sesamum indicum linn seeds with Rosa damascena mill oil in uncomplicated pelvic inflammatory disease using machine learning
Source: Front Chem. 2024 Apr 2;12:1361980. doi: 10.3389/fchem.2024.1361980 (PMC11018920; doi:10.3389/fchem.2024.1361980)
Supplement: Supplementary file 1 [file Table1.docx]

**Table S1.** Taxonomy and physicochemical parameters of *Sesamum indicum* L. seeds, *Rosa damascena* Mill flower and Rose oil

| 1. Taxonomy and Physicochemical Parameters of *Sesamum indicum* L. Seeds and *Rosa damascene* Mill Flower | | | |
| --- | --- | --- | --- |
| Taxonomy | *Sesamum indicum* L. seeds | *Rosa damascena* Mill flower | **Reference** |
| Kingdom | Plantae | Plantae |  |
| Sub-Kingdom | [Viridiplantae](https://www.itis.gov/servlet/SingleRpt/SingleRpt?search_topic=TSN&search_value=954898) | [Viridiplantae](https://www.itis.gov/servlet/SingleRpt/SingleRpt?search_topic=TSN&search_value=954898) |  |
| Infra-Kingdom | [Streptophyta](https://www.itis.gov/servlet/SingleRpt/SingleRpt?search_topic=TSN&search_value=846494) | [Streptophyta](https://www.itis.gov/servlet/SingleRpt/SingleRpt?search_topic=TSN&search_value=846494) |  |
| Super Division | [Embryophyta](https://www.itis.gov/servlet/SingleRpt/SingleRpt?search_topic=TSN&search_value=954900) | [Embryophyta](https://www.itis.gov/servlet/SingleRpt/SingleRpt?search_topic=TSN&search_value=954900) | (Rosa damascena Mill, 2023; Sesamum indicum L., 2023) |
| Division | [Tracheophyta](https://www.itis.gov/servlet/SingleRpt/SingleRpt?search_topic=TSN&search_value=846496) | [Tracheophyta](https://www.itis.gov/servlet/SingleRpt/SingleRpt?search_topic=TSN&search_value=846496) |  |
| Subdivision | [Spermatophytina](https://www.itis.gov/servlet/SingleRpt/SingleRpt?search_topic=TSN&search_value=846504) | [Spermatophytina](https://www.itis.gov/servlet/SingleRpt/SingleRpt?search_topic=TSN&search_value=846504) |  |
| Class | [Magnoliopsida](https://www.itis.gov/servlet/SingleRpt/SingleRpt?search_topic=TSN&search_value=18063) | [Magnoliopsida](https://www.itis.gov/servlet/SingleRpt/SingleRpt?search_topic=TSN&search_value=18063) |  |
| Superorder | [Asteranae](https://www.itis.gov/servlet/SingleRpt/SingleRpt?search_topic=TSN&search_value=846535) | [Rosanae](https://www.itis.gov/servlet/SingleRpt/SingleRpt?search_topic=TSN&search_value=846548) |  |
| Order | [Lamiales](https://www.itis.gov/servlet/SingleRpt/SingleRpt?search_topic=TSN&search_value=31632) | Rosales |  |
| Family | [Pedaliaceae](https://www.itis.gov/servlet/SingleRpt/SingleRpt?search_topic=TSN&search_value=32869) | [Rosaceae](https://www.itis.gov/servlet/SingleRpt/SingleRpt?search_topic=TSN&search_value=29200) |  |
| Genus | [Sesamum](https://www.itis.gov/servlet/SingleRpt/SingleRpt?search_topic=TSN&search_value=32870) L. | [Rosa](https://www.itis.gov/servlet/SingleRpt/SingleRpt?search_topic=TSN&search_value=29201) L. |  |
| Species | *Sesamum indicum L.* | *Rosa damascena* Mill. |  |
| Physicochemical Parameters | | |  |
| Ash Value (%) | | |  |
| Total ash | 15.960 | 5.00 | (Anonymous, 1997, 2006) |
| Acid insoluble Ash | 1.250 | 1.65 |  |
| Water soluble Ash | 0.834 | 2.40 |  |
| Successive extractive and non-successive extractive value (%) of *sesame* seeds and rose petal respectively | | | |
| Petroleum Ether | 43.30 | 4.68 | (Anonymous, 1997; Fathima and Murthy, 2019) |
| Chloroform | 2.04 | 2.12 |  |
| Abs. Alcohol | 2.55 | 17.24 |  |
| Water | 6.06 | 13.16 |  |
| Loss on Drying at 105°C | 5.13% | - |  |
| Moisture content | *-* | 14.32 |  |
| Total Phenolics (mgGAE/g) | 4.54 to 7.32 | 233.56±7.25 | (Zhou et al., 2016) (Baydar and Baydar, 2013) |
| Total Flavonoids (mgCE/g) | 5.8 to 7.6 | - |  |
| Total Flavanols | *-* | 28.71 ± 0.81 mgCE/g |  |
| Total Flavanols | *-* | 50.04 ± 2.35 mgRE/g |  |
| 1. Physicochemical Parameters of Rose Oil made by Traditional Method | | | |
| Appearance  Colour  Solubility in Pet. Ether (40-60°)  Saponification value  Acid value  Hydroxyl value  Peroxide value  Ester value  Unsaponifiable matter  Refractive index  Weight per ml  Test   - Sesame oil - Cotton seed oil - Arachis oil | | Oily  Yellow  100%  193.4-194.8  5.6-6.3  19.9  39.8  187-189  2.30%  1.475  0.912g  Positive  Negative  Negative | (Anonymous, 1986) |
